# Why Sensors Fail in Biological Samples: Fouling, Blocking, Matrix Effects and Prevention Solutions

**DOI:** 10.3390/ijms27125176

**Published:** 2026-06-07

**Authors:** Nikola Lenar, Beata Paczosa-Bator

**Affiliations:** Faculty of Materials Science and Ceramics, AGH University of Krakow, Mickiewicza 30, PL-30059 Krakow, Poland

**Keywords:** biosensors, chemical sensors, biofouling, matrix effects, nonspecific adsorption, biomarker detection, antifouling coatings, drift compensation

## Abstract

Sensors and biosensors designed for biomarker detection in biological samples often suffer from performance loss caused by surface fouling, interface blocking, and matrix interference. Although these effects are frequently discussed separately, in real sensing systems they are strongly interconnected and they determine analytical reliability, especially in body fluids like serum, plasma, whole blood, sweat, and other complex media. This review provides a practical and mechanism-oriented overview of how these processes originate, how they differ, and how they ultimately lead to signal drift, reduced sensitivity, false-positive responses, and shortened sensor lifetime. We first discuss the molecular origins of interface failure, including protein adsorption, conditioning film formation, nonspecific binding, ionic strength effects, pH fluctuations, viscosity-related diffusion changes, and electroactive interferents. The impact of these phenomena is then compared across major sensing platforms, including electrochemical, potentiometric, optical, capacitive sensors, field-effect transistors and wearable biosensors. A central part of this review focuses on practical prevention strategies already employed in real biomarker sensing platforms. These include hydration-driven antifouling coatings, zwitterionic and hydrogel interfaces, post-immobilization blocking with bovine serum albumin, mercaptohexanol and ethanolamine, ionophore and membrane engineering in ion-selective electrodes, hydrophobic solid-contact layers for water-layer suppression, regeneration workflows, membrane and microfluidic pre-treatment, and AI-assisted drift correction. By combining advances in materials engineering, surface chemistry, sample handling, and algorithmic correction, this review highlights strategies to improve sensor stability in complex biological fluids. Overall, it offers a practical guide for developing next-generation low-fouling, drift-resistant, and self-correcting sensing systems for reliable biomarker analysis at the point of care.

## 1. Introduction

Sensors and biosensors have become essential analytical tools in modern biomarker detection, enabling rapid, selective, and point-of-care diagnostics across clinical, environmental, and wearable applications. To provide the analytical context, the term “sensor” refers to a device that converts a physicochemical change caused by the presence of a target analyte into a measurable signal, without necessarily relying on a biological recognition element [1]. In contrast, biosensors incorporate biomolecules such as enzymes, antibodies, nucleic acids, peptides, cells, or biomimetic receptors as the selective recognition interface. In the present review, the term “sensors” therefore includes chemical and physicochemical transducers used for biomarker detection, such as ion-selective, electrochemical, optical, and plasmonic platforms, whereas “biosensors” specifically refers to systems in which molecular recognition is mediated by biologically derived or bioinspired elements [2,3]. This direct coupling between the biorecognition layer and the physicochemical transducer is what gives biosensors their high selectivity, but it also makes them particularly vulnerable to interfacial instability and matrix-induced signal distortion.

Recent advances in electrochemical, optical, plasmonic, and microfluidic sensors and biosensors have enabled the sensitive detection of clinically relevant biomarkers, including proteins, nucleic acids and metabolites, in complex biofluids such as serum, plasma, urine, saliva, sweat, and interstitial fluid [4,5]. These developments are especially important for point-of-care (POC) diagnostics and continuous health monitoring, where fast response, low sample volume, and device portability are critical. However, despite remarkable improvements in sensitivity and transduction strategies, the translation of biosensors from proof-of-concept experiments in buffered media to reliable operation in real biological samples remains a major unresolved challenge.

One of the most persistent limitations is the combined effect of surface fouling, receptor blocking, and matrix-derived signal interference, which frequently causes signal drift, sensitivity loss, poor reproducibility, and false-positive or false-negative responses [6]. In real biological matrices, the sensor surface is continuously exposed to proteins, lipids, salts, metabolites, cellular debris, and other competing species that can adsorb onto the transducer or biorecognition layer. These interactions may block active recognition sites, alter local charge distribution, hinder mass transport, or generate parasitic signals, ultimately reducing analytical reliability. Such effects are particularly problematic in biomarker detection, where target analytes are often present at trace concentrations and small signal distortions may lead to clinically misleading conclusions.

Importantly, fouling and matrix interference are often treated as separate analytical problems, although in practical sensing and biosensing systems they are strongly interconnected. Surface fouling may originate from protein adsorption, oxidation-product deposition, membrane pore blockage, biofilm formation, or irreversible passivation of electroactive interfaces, while matrix interference additionally includes electroactive interferents, ionic strength fluctuations, pH changes, viscosity effects, and nonspecific refractive index shifts in optical systems [7]. Together, these processes affect receptor accessibility, charge transfer kinetics, transducer response, and baseline stability, thereby compromising both short-term analytical performance and long-term operational robustness.

Although many previous reviews have focused on antifouling materials, passivation layers, and low-fouling coatings, these topics are often discussed mainly from a surface chemistry perspective. In practical biomarker sensing, however, signal deterioration rarely results from surface adsorption alone. Instead, analytical failure usually emerges from the combined action of fouling, incomplete surface blocking, matrix interference, drift, and sensor aging, especially in serum, blood, sweat, and other complex biological fluids.

This review treats these effects as interconnected mechanisms rather than isolated problems. We show how protein adsorption, membrane clogging, nonspecific binding, ionic strength changes, pH fluctuations, viscosity effects, and electroactive interferents lead to specific failure modes across electrochemical, potentiometric, optical, SPR, capacitive, and wearable biosensors. We then connect these mechanisms with practical prevention strategies, including hydration-driven antifouling materials, blocking workflows based on BSA, mercaptohexanol and ethanolamine, regeneration protocols, microfluidic pre-treatment, and AI-assisted drift correction. The review also extends this issue to ion-selective potentiometric sensors, where signal deterioration originates from ion competition, membrane co-extraction, and water-layer formation, and where prevention relies on ionophore selection, lipophilic ionic additives, and hydrophobic solid-contact engineering. By combining molecular mechanisms with platform-specific examples and validated prevention strategies, this review provides a practical guide for designing more robust sensors and biosensors for reliable biomarker detection in real biological samples.

## 2. Mechanisms of Sensor Failure

### 2.1. Distinction Between Fouling, Surface Blocking, and Matrix Interference

Before discussing prevention strategies and sensor-specific failure pathways, it is important to clearly distinguish the three closely related phenomena that most frequently compromise biomarker sensing in real samples: fouling, surface blocking, and matrix interference.

**Figure 1:**
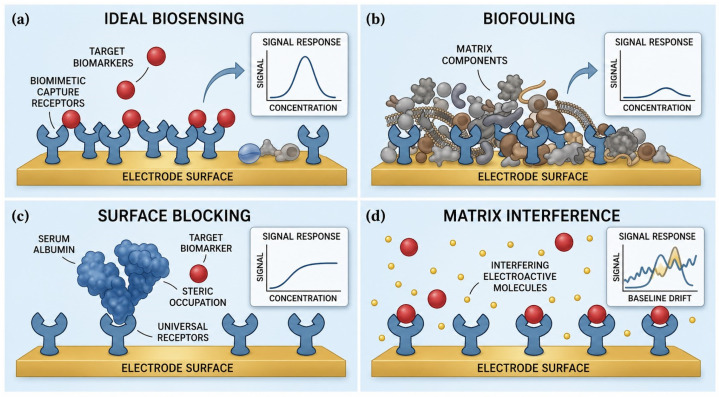
Schematic illustration of the three primary phenomena compromising sensor performance in complex biological samples. (**a**) Ideal Sensing: Target biomarkers bind specifically to biorecognition elements on a clean transducer surface, yielding a clear analytical signal. (**b**) Biofouling: Non-specific adsorption and gradual accumulation of proteins, lipids, and cellular debris form a passive layer, hindering mass transport and electron transfer. (**c**) Surface Blocking: Physical or steric occupation of active sites or pores by adsorbed macromolecules (e.g., serum albumin), directly preventing target access to receptors. (**d**) Matrix Interference: Signal distortion caused by soluble electroactive species or physicochemical fluctuations (e.g., pH, ionic strength) that alter the baseline without necessarily depositing on the interface. Prepared with the use of Gemini 3 Flash (Nano Banana 2 model).

In the context of sensors and biosensors, fouling can be defined as the nonspecific adsorption and gradual accumulation of unwanted species at the liquid–solid interface of the transducer or recognition layer, leading to physicochemical changes that deteriorate sensor performance [8,9,10]. In biological samples, foulants typically include proteins, lipids, nucleic acids, cells, extracellular vesicles, and oxidation by-products, which can form a passive layer on the sensing interface. This layer may hinder electron transfer, reduce receptor accessibility, alter interfacial wettability, and disturb mass transport of the target analyte [11]. Fouling is therefore a dynamic and progressive process, often becoming more severe with prolonged contact time, repeated measurements, or continuous monitoring. A schematic illustration of this phenomenon is shown in Figure 1b, with Figure 1a representing the ideal, undisturbed sensing condition.

Although often used interchangeably with fouling, surface blocking represents a more specific phenomenon. Here, the key event is the physical or steric occupation of catalytically active sites, ion-selective membrane pores, or biorecognition elements by adsorbed species, directly preventing target binding or signal generation (see Figure 1c) [8,12]. In other words, while fouling describes the broader accumulation process, surface blocking emphasizes the functional consequence of interface occupation. For example, serum albumin adsorbed on an SPR gold surface may not only contribute to general fouling but can also directly mask immobilized antibodies, reducing effective biomarker capture. Similarly, in electrochemical sensors, macromolecular adsorption at the membrane surface may obstruct ion exchange pathways, leading to slower equilibration and signal drift.

A third and partially independent issue is matrix interference, which refers to signal distortion caused by endogenous sample components that influence the analytical response without necessarily adsorbing onto the sensor surface [10]. Unlike fouling, matrix interference may arise from soluble electroactive molecules, ionic strength fluctuations, pH shifts, viscosity changes, nonspecific refractive index variations, or competing ligands present in the sample. Typical examples include ascorbic acid, uric acid, dopamine metabolites, interfering ions, and highly abundant plasma proteins that alter local physicochemical conditions. These species may generate overlapping electrochemical signals, change membrane selectivity, perturb binding equilibria, or modify optical baselines, even when no permanent surface deposition occurs. Schematically, interferences are presented in Figure 1d.

Importantly, these three phenomena are analytically distinct but mechanistically interconnected. Fouling frequently evolves into surface blocking when adsorbed macromolecules progressively cover active sensing regions. At the same time, matrix interference can accelerate fouling by promoting protein aggregation, changing ionic shielding, or altering biomolecular conformation at the interface. In real biofluids, these processes rarely occur in isolation. Instead, they form a coupled cascade in which molecular adsorption, local physicochemical changes, and signal artifacts amplify one another, ultimately leading to drift, loss of selectivity, poor reproducibility, and clinically misleading results.

This mechanistic distinction is particularly important for biomarker detection, where the target signal is often several orders of magnitude lower than the concentration of potentially interfering matrix components. Biofouling, surface blocking, and matrix interferences are universal phenomena affecting both chemical sensors and biosensors; however, their impact depends on the sensing principle. In biosensors, these processes primarily compromise the biorecognition layer by masking binding sites or altering biomolecular activity, whereas in chemical sensors, they mainly disrupt the transducer function by limiting surface accessibility or altering signal generation pathways. Matrix effects such as pH, ionic strength, and electroactive species influence both classes, but they perturb either molecular recognition or physicochemical signal conversion depending on the system architecture. These fundamental differences and similarities are summarized in Table 1, which highlights how identical interfacial phenomena manifest differently depending on whether signal generation is governed by molecular recognition (biosensors) or direct physicochemical transduction (chemical sensors).

### 2.2. Molecular Origins of Fouling and Blocking

#### 2.2.1. Conditioning Film Formation

**Figure 2:**
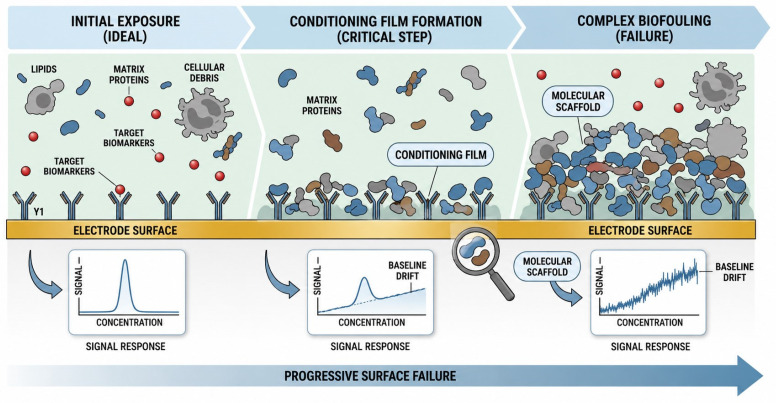
Molecular dynamics of the liquid–solid interface failure over time. (Stage 1) Initial Exposure: Rapid transport of abundant matrix components toward the sensor surface. (Stage 2) Conditioning Film Formation: proteins undergo conformational rearrangements, creating a “molecular scaffold” at the interface. This leads to early baseline drift and altered local wettability. (Stage 3) Complex Biofouling: The conditioning film promotes secondary accumulation of larger biomolecular aggregates and cellular debris, resulting in a thick, insulating layer. This progressive cascade ultimately leads to severe signal suppression, loss of sensitivity, and irreversible sensor failure. Prepared with the use of Gemini 3 Flash (Nano Banana 2 model).

The initiation of fouling and surface blocking is fundamentally governed by molecular interactions occurring at the sensor–sample interface, where biomolecules and endogenous matrix components compete for access to the transducer or biorecognition layer. In biological samples, the earliest stage of interface failure often begins within seconds of exposure, when abundant proteins such as albumin, fibrinogen, immunoglobulins, and lipoproteins rapidly adsorb onto the sensing surface [13]. This initial adsorption layer, often referred to as a conditioning film, serves as a molecular scaffold for secondary accumulation of additional biomolecules, cellular debris, or oxidation by-products [14], thereby accelerating progressive signal deterioration. The schematic representation of this phenomenon is presented in Figure 2.

#### 2.2.2. Physiochemical Mechanisms of Fouling

The adsorption process is driven by a combination of:

- hydrophobic interactions;
- electrostatic attraction;
- hydrogen bonding;
- van der Waals forces;
- steric confinement effects [8,15].

Hydrophobic surfaces, such as many carbon-based electrodes and polymeric membranes, are particularly susceptible to protein adsorption. This occurs because biomolecules tend to lower their interfacial free energy by orienting their nonpolar regions toward the surface. After adsorption, proteins can undergo conformational changes that increase their residence time and reduce the likelihood of desorption. As a result, initially reversible adsorption may become quasi-irreversible, leading to persistent surface fouling and long-lasting signal artifacts.

Electrostatic interactions further modulate adsorption selectivity and kinetics. Charged sensor interfaces can attract oppositely charged proteins, metabolites, nucleic acids, or extracellular vesicles, especially under low ionic strength conditions where electrostatic screening is limited [12,15,16,17]. In potentiometric and ion-selective systems, such effects may additionally perturb the local ion distribution at the membrane surface, altering phase-boundary equilibria and leading to potential drift. Similarly, in impedimetric biosensors, the accumulation of charged macromolecules near the interface may produce significant nonspecific impedance changes that mimic target recognition events.

A particularly important molecular mechanism in electrochemical sensors is the formation of insulating reaction-product films. Electroactive metabolites such as dopamine, catecholamines, phenols, uric acid derivatives, and certain pharmaceuticals may undergo oxidation followed by polymerization or covalent deposition on the electrode surface [8,18]. Unlike protein adsorption, this process creates a chemically transformed passivation layer that directly suppresses electron transfer kinetics and reduces the effective electroactive area. Such deposition-induced fouling is one of the major reasons why sensors performing well in simple buffers often lose sensitivity in serum, plasma, or tissue-derived samples.

Surface topography and roughness also play a major mechanistic role. Nanostructured and porous interfaces provide enhanced sensitivity due to their high surface area, but they may simultaneously create nanoscale trapping sites for proteins, lipids, and aggregates, increasing susceptibility to irreversible fouling [13]. This is especially relevant in nanocomposite electrodes, porous gold SPR surfaces, and hydrogel-coated biosensors, where the same architecture that improves signal amplification may also facilitate multivalent adsorption and hinder complete regeneration.

In optical and plasmonic biosensors, even weak molecular adsorption can lead to pronounced analytical consequences. Nonspecific protein accumulation near the metal surface changes the local refractive index and mass loading, which may shift the baseline and generate false binding signals [12]. Importantly, these artifacts do not always reflect direct receptor blockage but may still significantly distort biomarker quantification, especially in label-free assays.

These early interfacial events rarely occur in isolation, but instead evolve into coupled processes that progressively reduce receptor accessibility, alter ion or electron transport, destabilize the baseline, and amplify matrix-dependent signal artifacts in downstream sensing measurements. Understanding these interactions at the molecular level is essential for rationally designing fouling prevention strategies that go beyond passive surface coatings [18,19].

## 3. Matrix Interferences in Biological Samples

While Section 2 explained the molecular and physicochemical causes of interface instability, the real analytical impact becomes clearer when we look at individual components of biological samples. In practical biomarker detection, the sensor signal depends not only on recognition of the target molecule, but also on the presence of highly abundant endogenous species that can cause nonspecific adsorption, electrochemical cross-talk, baseline drift, or slower mass transport [20,21]. These effects are especially important in serum, plasma, whole blood, saliva, urine, sweat, and interstitial fluid, where sample composition can vary significantly between patients, disease states, and even sample collection or storage conditions.

To provide a practical approach for sensor and biosensor design, this section discusses the major classes of biological interferents responsible for analytical failure, including proteins, lipids, lipoproteins, electroactive metabolites, electrolyte fluctuations, rheological changes, and preanalytical artifacts.

### 3.1. Proteins and Lipids Interferents

Among all biological matrices, serum and plasma present some of the most challenging environments for biomarker sensing, primarily due to their extremely high content of proteins and amphiphilic lipid carriers [19,20]. Albumin, immunoglobulins, fibrinogen, transferrin, and complement proteins are typically present at concentrations many orders of magnitude higher than target biomarkers, making nonspecific adsorption statistically favored even on highly selective sensing interfaces. As a result, these macromolecules rapidly form a surface conditioning layer that modifies the local charge distribution, hydration structure, and receptor accessibility.

Albumin is particularly important because of its abundance, conformational flexibility, and strong tendency to adsorb onto metallic, carbonaceous, and polymeric interfaces [21]. Once adsorbed, it may mask biorecognition elements, alter electron transfer pathways, and create steric barriers that reduce analyte diffusion toward the sensing surface. In potentiometric sensors, protein adsorption may additionally perturb the local ion activity profile at the membrane boundary, leading to unstable phase-boundary potentials and signal drift.

Lipids and lipoproteins introduce a second level of complexity. Low-density lipoproteins (LDL), high-density lipoproteins (HDL), triglyceride-rich particles, and membrane fragments possess both hydrophobic and charged domains, which facilitate strong interaction with nanostructured and plasmonic surfaces [19]. Their adsorption can substantially alter local dielectric properties and interfacial refractive index, making them particularly problematic in SPR, LSPR, and label-free optical biosensors. In electrochemical systems, amphiphilic lipid layers may partially passivate the electrode surface and slow down charge-transfer kinetics.

Importantly, serum proteins and lipoproteins rarely act independently. Instead, they frequently co-adsorb into a mixed biomolecular corona-like layer, where proteins stabilize lipid-rich aggregates and lipoproteins enhance secondary protein retention. This cooperative interfacial restructuring significantly increases the risk of false-positive binding, baseline elevation, and long-term regeneration failure [19,21]. Such effects are especially pronounced in repeated-use biosensors and in continuous sensing systems exposed to flowing biological fluids.

### 3.2. Small-Molecule Interferents

Beyond macromolecular fouling caused by proteins and lipoproteins, biological fluids contain a wide range of low-molecular-weight electroactive species that significantly distort electrochemical biosensor responses. These compounds are particularly problematic because they are often present at relatively high and fluctuating concentrations and may exhibit redox potentials overlapping with those of target analytes. As a result, they can generate false-positive signals, baseline elevation, and reduced analytical specificity, especially in amperometric and voltammetric sensing platforms [22,23].

Among the most studied interferents are uric acid and ascorbic acid, which are both abundant in serum and interstitial fluid. Uric acid is an end product of purine metabolism, while ascorbic acid (vitamin C) is a key antioxidant present in many physiological fluids. Both compounds are readily oxidizable at commonly used electrode materials, including carbon-based and metal-modified surfaces. Their simultaneous oxidation can lead to overlapping voltammetric peaks, making it difficult to resolve target-specific signals without additional separation strategies or selective surface modifications [22].

A related and highly relevant issue is glucose cross-talk, which is particularly critical in the context of diabetes monitoring and continuous glucose sensing. In non-enzymatic glucose sensors, oxidation currents attributed to glucose may be significantly affected by coexisting electroactive species, especially uric acid, ascorbic acid, and acetaminophen metabolites. Even in enzymatic glucose biosensors, interference may arise indirectly through changes in local oxygen concentration, pH shifts caused by metabolic activity, or electrode surface fouling that alters enzyme accessibility and electron transfer efficiency [23]. These effects can result in signal drift and calibration instability during long-term operation.

Importantly, the impact of small-molecule interferents is strongly dependent on the electrode material, surface functionalization, and applied potential window. Nanostructured materials may enhance sensitivity but simultaneously increase the density of active sites where nonspecific oxidation can occur. Similarly, catalytic surfaces designed to improve electron transfer may unintentionally lower selectivity by facilitating the oxidation of multiple species at similar potentials.

In addition to direct electrochemical cross-reactivity, these small molecules can also influence interfacial charge distribution and double-layer structure, thereby indirectly affecting potentiometric and impedimetric measurements. Changes in local ionic composition and redox balance may shift baseline stability, especially in low-buffer-capacity environments such as sweat or interstitial fluid.

### 3.3. Electrolytes, pH Variation, and Ionic Strength Effects

Beyond macromolecular and electroactive molecular interferents, the electrolyte composition of biological samples can also significantly distort sensor responses. Variations in the concentration of major ions such as Na^+^, K^+^, Cl^−^, Ca^2+^, Mg^2+^, phosphate, bicarbonate, and ammonium directly influence the physicochemical environment at the sensor interface, often leading to substantial baseline shifts and reduced selectivity [24]. These effects are especially pronounced in potentiometric and ion-selective sensing systems, where the analytical signal is inherently governed by phase-boundary equilibria and local ion activity rather than total concentration.

In serum and plasma, physiological ionic strengths are typically high, causing strong electrostatic screening and compression of the electrical double layer. This may suppress charge-based recognition events, reduce sensitivity in field-effect and potentiometric biosensors, and introduce deviations from ideal Nernstian behavior. In wearable biofluids such as sweat and interstitial fluid, ionic strength may fluctuate dynamically with hydration status, perspiration rate, and metabolic activity, making stable calibration even more difficult.

pH variation introduces an additional layer of complexity. Changes in proton concentration can alter the protonation state of membrane components, receptor ligands, surface functional groups, and biorecognition elements. As a consequence, the affinity of ionophores, aptamers, peptides, antibodies, and enzyme-based recognition layers may shift significantly even when the target biomarker concentration remains constant. In potentiometric systems, local pH fluctuations can directly perturb the membrane potential, while in enzymatic biosensors, pH changes may modify catalytic turnover and product generation rates.

Electrolyte-induced perturbations are also critically linked to reference electrode instability and junction potential artifacts. Differences between calibration buffers and real biological matrices may create liquid-junction potentials, especially when the chloride concentration, protein content, or viscosity differs substantially between solutions. These hidden potential offsets are a frequent source of poor reproducibility and sample-to-sample variability in clinical measurements.

Biological fluids such as serum and plasma contain a high concentration of dissolved ions (e.g., Na^+^, Cl^−^), which strongly influence how electrical signals behave near sensor surfaces. When a charged molecule binds to a receptor on an electrochemical or field-based biosensor, it generates an electrical perturbation that can, in principle, be detected. However, in high-salt environments, surrounding ions rapidly redistribute and shield these charge effects, a phenomenon known as Debye screening [25]. As a result, the electrical influence of a binding event decays very quickly with distance from the surface, and only events occurring extremely close to the sensor interface can be effectively detected. This strong attenuation of electrostatic interactions in physiological media reduces the effective sensing range of charge-sensitive biosensors and is a key limitation for aptasensors, immunosensors, and potentiometric devices operating in undiluted biological samples.

To summarize, electrolyte composition, pH fluctuations, and ionic strength effects act as fundamental matrix-dependent modulators of sensor response, influencing ion activity, surface charge distribution, receptor conformation, and electrostatic sensing range. These effects are particularly critical in miniaturized and wearable platforms, where even small changes in local sample composition may propagate into substantial analytical drift.

### 3.4. Viscosity, Diffusion Limitations, and Hemolysis Artifacts

Beyond chemical interferents, the physical properties and preanalytical quality of biological samples can also distort sensor performance. Increased sample viscosity slows analyte diffusion toward the sensing interface, prolongs equilibration, and may lead to underestimated biomarker levels, particularly in microfluidic, SPR, impedimetric, wearable, and ion-selective sensors, where transport can become rate-limiting [26,27]. This effect is especially relevant in protein-rich serum, mucus-containing saliva, wound exudates, and paper-based systems, where altered rheology changes diffusion distance, capillary flow, and membrane hydration.

Another important source of error is hemolysis, which releases hemoglobin, intracellular ions, enzymes, membrane fragments, and redox-active metabolites into serum or plasma [28]. These components can distort electrochemical, optical, and ion-selective readouts through background absorbance, oxidation currents, nonspecific adsorption, or false elevation of ions such as K^+^.

Together, viscosity-related diffusion limits and hemolysis represent practical matrix artifacts that are especially critical in miniaturized and rapid-response biosensors.

## 4. Transducer-Specific Failure Mechanisms

Although fouling and matrix interference arise from common physicochemical principles, their analytical consequences strongly depend on the signal transduction mechanism [6,7]. The same matrix component may therefore produce different artifacts in potentiometric, amperometric, impedimetric, optical, or field-effect sensors. Understanding these platform-specific failure pathways is therefore essential for selecting prevention strategies that match transducer physics.

### 4.1. Potentiometric Sensors

In potentiometric and ion-selective sensors, matrix-induced failure mainly arises from changes in ion activity, membrane surface fouling, water-layer formation, and reference electrode mismatch [24,29,30,31,32]. Adsorption of proteins, lipids, and cellular debris onto the membrane can alter local hydration and phase-boundary equilibrium, leading to baseline drift, hysteresis, and deviations from ideal Nernstian response. In solid-contact devices, buried water layers and hydration-driven instability further amplify long-term drift, particularly in wearable and continuously operating sensors. Because these systems respond to ion activity rather than total concentration, fluctuations in ionic strength, osmolarity, and viscosity may additionally produce misleading calibration shifts.

### 4.2. Voltammetric/Amperometric Sensors

In voltammetric and amperometric platforms, failure is dominated by electrode passivation, oxidation-product deposition, and electroactive cross-talk [33,34]. Matrix components such as proteins, lipids, uric acid, ascorbic acid, and drug metabolites may block catalytic sites or generate overlapping redox currents, leading to suppressed peaks, elevated background current, and false-positive responses. These effects are especially severe in continuous and wearable amperometric sensors, where prolonged electrode polarization accelerates irreversible surface poisoning.

### 4.3. Impedimetric and Capacitance-Based Biosensors

Impedimetric and capacitive biosensors are particularly vulnerable because both specific recognition and nonspecific adsorption can generate similar electrical signatures [35,36]. Proteins, extracellular vesicles, and lipid-rich biomolecules may increase charge-transfer resistance or alter the dielectric thickness, producing false-positive impedance or capacitance changes. Variations in ionic strength and pH further shift equivalent-circuit parameters independently of analyte concentration, making advanced circuit validation and low-fouling interfaces especially important.

### 4.4. Optical and Plasmonic Sensors

In optical and plasmonic sensors, the main failure pathways include nonspecific mass loading, bulk refractive-index fluctuations, and incomplete regeneration of the sensing surface [19,37]. Proteins, lipoproteins, and hemolysis products may adsorb onto gold or nanoplasmonic interfaces and generate resonance shifts that closely resemble true binding events. Because these systems directly respond to refractive-index changes, even small differences in sample protein content, osmolarity, or temperature may produce substantial baseline drift.

### 4.5. BioFETs and Wearable Biosensors

Field-effect transistor biosensors and wearable bioelectronic platforms are especially sensitive to electrostatic screening, soft-interface adsorption, and threshold-voltage drift [38,39]. In physiological fluids, the high concentration of ions compresses the Debye length, meaning that electrical signals from the recognition layer are rapidly screened and cannot effectively reach the semiconductor channel. At the same time, proteins, salts, and hydration layers may progressively alter local surface charge and dielectric properties, leading to drift, hysteresis, and increased noise. In flexible wearable devices, these artifacts are further amplified by repeated mechanical deformation and hydration–dehydration cycles.

These examples show that the same matrix component can affect different sensor platforms in very different ways. As presented in Table 2, each sensing modality has its own main failure mechanisms and characteristic signal artifacts, from ion-activity changes in potentiometric sensors to refractive-index drift in plasmonic systems and charge screening in BioFETs.

This comparison shows that matrix effects should always be considered in the context of the specific transduction mechanism, which is essential when choosing the most suitable prevention strategy for reliable biomarker detection in real biological samples.

## 5. Prevention Strategies

Because failure mechanisms differ across sensing platforms, prevention strategies must target the specific physicochemical source of signal distortion rather than only the final analytical artifact alone [40,41]. No single solution is universally effective, as matrix-induced errors may arise from nonspecific adsorption, oxidation-product deposition, electrolyte screening, transport limitations, regeneration failure, or baseline drift. For this reason, robust sensor design increasingly relies on multilayered approaches that combine surface chemistry, interface engineering, device architecture, sample pre-treatment, and data-driven correction.

At the materials level, common strategies include hydration-based antifouling coatings, zwitterionic and hydrogel interfaces, selective membranes, and nanoporous barriers that reduce protein and lipid adsorption while maintaining analyte transport [40,41]. At the device level, improved membrane composition, stable solid contacts, regenerable surfaces, and microfluidic sample control help minimize long-term drift and matrix burden [41,42]. When physical fouling prevention is not sufficient, informatic tools such as baseline tracking, adaptive calibration, and machine-learning-assisted drift correction provide an additional layer of robustness [40].

The following subsections discuss these prevention strategies in detail, moving from surface-level antifouling approaches to device-level stabilization and data-driven compensation methods.

### 5.1. Surface Antifouling Materials and Hydrated Interfaces

#### 5.1.1. General Principles of Hydration-Based Antifouling

Recent studies show that hydration-driven interfaces, particularly PEGylated layers, zwitterionic peptides, and conductive hydrogels, can substantially improve analytical reliability in serum and other protein-rich media by suppressing nonspecific adsorption while preserving target accessibility [43,44,45,46,47,48].

**Figure 3:**
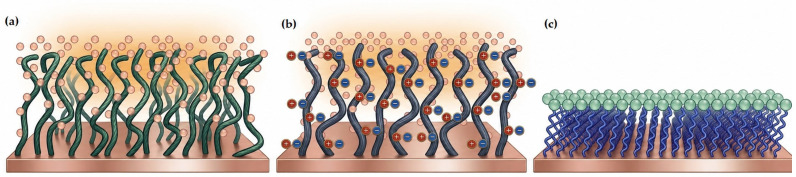
Hydration-based antifouling surface architectures for sensor protection. Comparison of (**a**) hydrophilic polymer brushes, (**b**) zwitterionic polymer brushes, and (**c**) self-assembled monolayers (SAMs), illustrating differences in hydration shell formation, steric exclusion, and molecular packing at gold transducer surfaces. Adapted from Chen et al. [49]. Prepared with the use of Gemini 3 Flash (Nano Banana 2 model).

The role of surface hydration and chain flexibility in antifouling performance is illustrated in Figure 3, comparing hydrophilic polymers, zwitterionic coatings, and self-assembled monolayers. Figure 3 summarizes the main hydration-based antifouling surface architectures used to protect sensing interfaces. As shown, hydrophilic polymer brushes such as PEG create a diffuse hydration shell through flexible surface-grafted chains, generating steric and water-mediated repulsion against proteins and other foulants. In contrast, zwitterionic polymer brushes (e.g., polySBMA) produce a more localized and strongly bound hydration layer through balanced positive and negative charges, which is particularly effective in resisting nonspecific adsorption from complex biofluids. Self-assembled monolayers (SAMs) represent a thinner and more ordered alternative, where densely packed molecular layers provide steric exclusion and limit the access of larger biomolecules to the transducer surface. Together, these architectures illustrate how controlling interfacial hydration and steric accessibility can significantly improve antifouling performance while maintaining analyte transport to the sensing layer.

Hydration-based antifouling materials include both surface-grafted polymer brushes and bulk hydrogel networks, which differ in architecture but share the ability to resist nonspecific adsorption through steric and water-mediated repulsion [50]. While polymer brushes provide an excellent 2D anti-fouling interface, 3D hydrogel matrices offer a versatile architecture for enhancing the ionic and mechanical performance of sensors and biosensors.

#### 5.1.2. Hydrogel and Peptide-Based Antifouling Matrices

One of the strongest examples of hydrogel application is the zwitterionic peptide hydrogel electrochemical biosensor for prostate-specific antigen (PSA) reported by Du et al. [43]. In this system, a self-assembled zwitterionic peptide hydrogel (CFEFKFC) was integrated with a PEDOT/AuNP electrochemical platform for PSA detection in human serum. The hydrogel created a highly hydrated antifouling layer that effectively prevented nonspecific adsorption of proteins and cells, enabling reliable sensing in real serum with a linear response range from 0.1 ng mL^−1^ to 100 ng mL^−1^ and a LOD of 5.6 pg mL^−1^.

The developed HER2 biosensor exhibited a linear detection range from 0.1 ng mL^−1^ to 1.0 μg mL^−1^ with a detection limit of 45 pg mL^−1^ in human serum. Wang et al. developed an antifouling peptide hydrogel-based electrochemical biosensor for HER2 detection in human serum, where the peptide hydrogel served as both the antifouling interface and antibody-supporting scaffold [44]. The highly hydrated 3D matrix suppressed serum protein adsorption while preserving diffusion of the HER2 target toward the sensing interface, illustrating how hydrogel porosity and water retention can decouple antifouling from mass transport limitations.

One of the strategies is PEG-assisted hydration shielding. Yang et al. demonstrated this concept in an electrochemical HER2 biosensor for complex biological media, where PEG was combined with a designed recognizing peptide on PEDOT/AuNP electrodes [45]. The dual antifouling design significantly improved signal quality in serum-containing samples, enabling HER2 detection over a wide linear range from 1.0 pg mL^−1^ to 1.0 μg mL^−1^ with an ultralow detection limit of 0.44 pg mL^−1^.

The PEG concept was pushed further by Shi et al., who reported a super-antifouling electrochemical biosensor for human Immunoglobulin G (IgG) detection in complex biofluids based on a PEGylated multifunctional peptide [46]. Here, PEG was not only used as a hydration-promoting layer but also integrated directly into the multifunctional recognition peptide architecture. The system achieved a wide linear detection range from 1 pg mL^−1^ to 500 ng mL^−1^ together with a sub-pg mL^−1^ detection limit in serum.

Another highly relevant practical direction is the use of low-fouling α/β-peptide interfaces with enhanced enzymatic stability. Zhao et al. showed that α/β-peptide-based electrochemical biosensors maintain reliable biomarker detection in human serum, overcoming one of the key limitations of classical peptide antifouling layers [47].

Most recently, conductive hydrogel architectures have emerged as an especially powerful class of multifunctional antifouling interfaces. A double-conductive hydrogel electrochemical biosensor by Geng et al. demonstrated that hydrogels can provide simultaneous antifouling protection, charge transport, and signal amplification in complex serum media [48].

**Figure 4:**
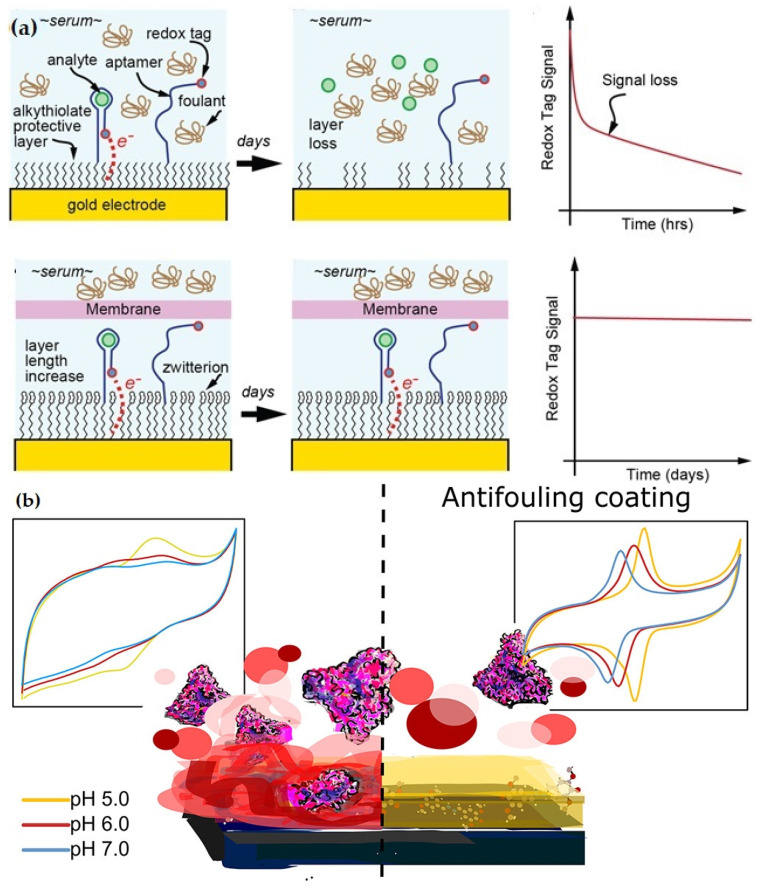
Long-term signal preservation in electrochemical biosensors through antifouling surface engineering. (**a**) Schematic illustration of fouling-induced degradation in an aptamer-based electrochemical sensor operating in serum, showing progressive monolayer loss, aptamer destabilization, and decay of the redox-tag signal over time. Reproduced from Young et al. [50]. (**b**) Incorporation of antifouling strategies, such as protective membranes or zwitterionic coatings, stabilizes the sensing interface. These antifouling layers act as permselective barriers that reduce biofouling, minimize signal distortion, and enable stable, well-defined electrochemical responses across varying pH conditions. Reproduced from Jarosinska et al. [9].

The long-term analytical consequences of biofluid-induced fouling, together with the stabilizing effect of protective membranes and zwitterionic antifouling monolayers, are illustrated schematically in Figure 4a. The effect of antifouling surface coatings on electrochemical signal stability is also illustrated schematically in Figure 4b, highlighting the suppression of nonspecific adsorption and preservation of redox activity across varying pH conditions.

Unlike passive polymer brushes, conductive hydrogels allow the interface to remain electrochemically active while minimizing nonspecific protein deposition, making them highly promising for wearable and continuous biomarker sensing. These real-world examples show that the most successful antifouling strategies are no longer simple blocking coatings, but rather multifunctional hydrated interfaces that integrate fouling resistance, bioreceptor stabilization, conductivity, and selective mass transport.

#### 5.1.3. Polymer Brushes, Zwitterions, and SAMs

Another highly effective hydration-based antifouling strategy involves surface-grafted polymer brushes, in which densely tethered polymer chains extend from the sensor surface to create a steric and strongly hydrated barrier against nonspecific adsorption. Unlike bulk hydrogels, polymer brushes form ultrathin nanometer-scale coatings that preserve direct transducer coupling while still resisting protein, lipid, and bacterial adhesion. Particularly important examples include oligo(ethylene glycol) methacrylate (OEGMA) and carboxybetaine-based brushes, which have shown excellent resistance to fouling from complex biological fluids including plasma, saliva, urine, and serum. Rodríguez-Emmenegger and co-workers demonstrated that such brushes maintain low nonspecific adsorption even after biofunctionalization, making them highly attractive for SPR, electrochemical, and BioFET biosensors operating in real biological matrices [51,52,53]. In addition to suppressing protein adsorption, polymer brushes can reduce bacterial attachment and delay early biofilm formation, which is especially relevant for wearable and long-term implanted sensing interfaces.

Beyond generic surface chemistries, these principles are increasingly translated into sensor-specific protective coatings tailored to the failure pathways of individual transduction platforms.

#### 5.1.4. Electrosynthesized Permselective Polymer Coatings

Electrosynthesized permselective polymer films represent another important strategy for preventing matrix interference in electrochemical biosensors operating in complex biological and food-related samples. Unlike hydration-based antifouling interfaces, these coatings primarily function through electrostatic and size-exclusion mechanisms that selectively suppress the access of electroactive interferents while preserving analyte transport to the transducer surface. Particularly important examples include overoxidized polypyrrole and related electropolymerized non-conducting films, which can act as highly stable permselective barriers against compounds such as ascorbic acid, uric acid, dopamine, and other redox-active interferents commonly present in biological matrices [54,55,56].

A representative example was reported by Ciriello and Guerrieri, who developed an amperometric choline oxidase biosensor based on an overoxidized polypyrrole film deposited on a platinum electrode for phospholipase D activity analysis [54]. The overoxidized polymer layer exhibited strong rejection properties toward interfering electroactive compounds while preserving choline transport and hydrogen peroxide detection. The system demonstrated good analytical stability together with practical applicability in complex matrices. Related studies further demonstrated that electrosynthesized permselective polymer architectures can be integrated with enzyme immobilization and dual-electrode biosensing strategies to improve selectivity and suppress signal cross-talk in biological samples [55,56]. Unlike passive blocking layers, these electrochemically generated coatings combine permselective transport control with direct integration into the transducer fabrication process, making them particularly attractive for miniaturized amperometric biosensors and point-of-care analytical platforms.

In addition to hydration-driven antifouling, electrostatic and permselective coatings represent another important strategy for preserving sensor performance in complex biological matrices. A classic example is Nafion, a negatively charged sulfonated fluoropolymer widely used to suppress interference from anionic species such as ascorbate, urate, and negatively charged proteins while maintaining permeability toward selected analytes and redox mediators. Due to its combined ion-exchange and permselective properties, Nafion has been extensively applied in electrochemical biosensors to reduce matrix-induced signal distortion and improve operational stability in serum and physiological fluids [57].

Conducting polymer systems such as PEDOT:PSS additionally provide mixed ionic-electronic conductivity together with enhanced interfacial stability and reduced nonspecific adsorption. In biosensing applications, PEDOT:PSS coatings help preserve transducer sensitivity by stabilizing charge transfer at the biointerface while maintaining compatibility with hydrated antifouling architectures. These electrostatic and conductive polymer interfaces are particularly valuable in wearable and continuous sensing systems, where long-term exposure to protein-rich media can otherwise rapidly deteriorate signal quality [58].

#### 5.1.5. Transducer-Specific Protective Coatings for ISE

**Figure 5:**
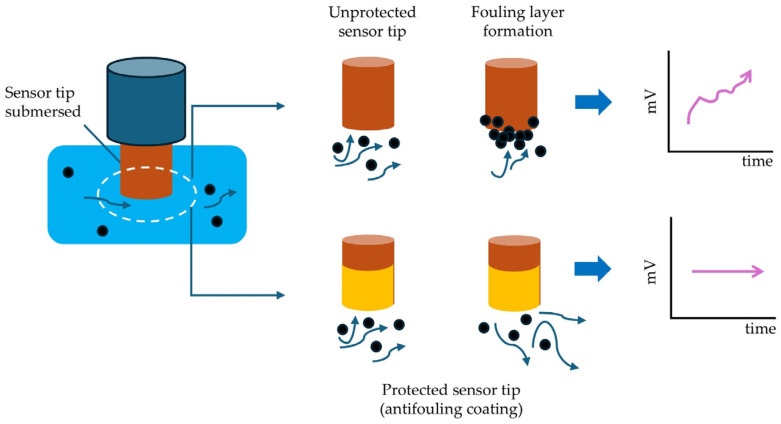
Schematic representation of fouling-induced drift in ion-selective electrodes (ISEs). Unprotected membrane tips progressively accumulate foulants, leading to time-dependent potential drift, whereas antifouling-coated sensor tips preserve a stable potentiometric response. Reproduced from Rinn et al. [59].

A practical example of antifouling coating design is found in ion-selective electrodes (ISEs) used for long-term monitoring. In these systems, external fouling of the exposed membrane tip may become a major source of drift during continuous operation in biological and environmental samples. Adsorption of proteins, lipids, biofilm precursors, and particulate matter creates an additional diffusion and hydration barrier that gradually alters local ion exchange, leading to progressive potential drift and delayed stabilization [59]. Recent ISE-focused studies show that protective hydrophilic or low-adhesion coatings, including silicone hydrogels, fluoropolymer foul-release layers, zwitterionic polymer coatings, and TiO_2_-based superhydrophilic surfaces, can strongly suppress fouling-layer formation and preserve a stable millivolt response over time compared with unprotected membranes (Figure 5). This strategy is particularly relevant for wearable, epidermal, and long-term immersed ISEs.

Although nanostructured and porous interfaces significantly enhance analytical sensitivity by increasing electroactive surface area and receptor loading, excessive roughness and nanoscale porosity may also promote trapping of proteins, lipids, extracellular vesicles, and biomolecular aggregates within the interfacial architecture. In complex biological matrices, this may lead to accelerated irreversible fouling, restricted mass transport, and progressive signal instability. Importantly, the current literature does not define a universal roughness threshold at which sensitivity gains consistently outweigh fouling risks, as this balance depends strongly on nanostructure geometry, wettability, pore accessibility, and sample composition. Consequently, optimal nanointerface design increasingly focuses on balancing surface-area enhancement with controlled mass transport and antifouling accessibility.

### 5.2. Surface Blocking After Bioreceptor Immobilization

While antifouling materials reduce nonspecific adsorption at the design level, most biosensors based on specific molecular recognition still require additional surface passivation to block remaining active sites, exposed regions of the transducer, and gaps between immobilized sensing molecules. This is especially important in gold self-assembled monolayer (SAM) electrochemical sensors, aptasensors, and immunosensors based on 1-ethyl-3-(3-dimethylaminopropyl)carbodiimide (EDC) and N-hydroxysuccinimide (NHS) coupling chemistry, where incomplete surface blocking or insufficient gap filling can lead to false-positive signals in serum and whole blood [60].

One of the most established post-immobilization strategies is ethanolamine quenching of residual NHS esters, especially after EDC/NHS coupling on carboxyl-terminated SAMs or polymeric biointerfaces. This step chemically deactivates unreacted activated esters that would otherwise bind proteins or serum interferents nonspecifically. In practical sensor fabrication, ethanolamine therefore acts as a reactive-site terminator, stabilizing the electrochemical or SPR baseline before exposure to complex media [60].

A second mechanistically distinct strategy is MCH (6-mercapto-1-hexanol) backfilling, which is especially important in gold-based DNA and aptamer biosensors. After immobilization of thiolated probes, MCH is routinely introduced to occupy uncovered gold domains, improve monolayer packing density, optimize probe orientation, and reduce direct adsorption of proteins or electroactive species [61,62]. This structural ordering effect is essential for minimizing charge-transfer leakage and signal variability in label-free electrochemical systems.

At the protein level, BSA remains the most widely used universal blocking agent in immunosensors and biosensors for serum analysis. By adsorbing onto residual exposed surface regions, BSA suppresses nonspecific attachment of serum proteins, lipoproteins, and extracellular vesicles that would otherwise generate background current, false refractive-index shifts, or impedance drift.

An example of BSA-based post-functionalization blocking in a real biomarker assay is the electrochemical immunosensor for carcinoembryonic antigen (CEA) in human serum [63]. After immobilization of the anti-CEA recognition layer on a biomaterial-modified glassy carbon electrode, the authors optimized the BSA blocking step (60 min) to suppress nonspecific adsorption from serum proteins and stabilize the ferri/ferrocyanide charge-transfer response. Importantly, the analytical performance strongly depended on this passivation stage, demonstrating that BSA remains indispensable even in modern nanostructured biomaterial interfaces.

Another example comes from a miRNA-21 electrochemical biosensor, where the authors used a combined MCH + BSA blocking workflow after immobilization of the DNA hairpin probe [64]. The MCH backfilled uncovered gold sites within the SAM, while BSA suppressed nonspecific macromolecular adsorption. Electrochemical impedance spectroscopy showed a further increase in charge-transfer resistance immediately after this dual blocking step, directly confirming successful passivation of residual surface defects.

A particularly valuable extension of classical blocking chemistry is the use of BSA-derived structural passivation layers, such as crosslinked BSA hydrogels, where the blocking reagent simultaneously acts as a low-fouling scaffold [65].

Mechanistically, post-functionalization blocking therefore fulfills three complementary and synergistic roles:

- chemical deactivation of reactive groups (ethanolamine);
- defect filling and SAM ordering (MCH);
- suppression of nonspecific protein adsorption (BSA).

**Figure 6:**
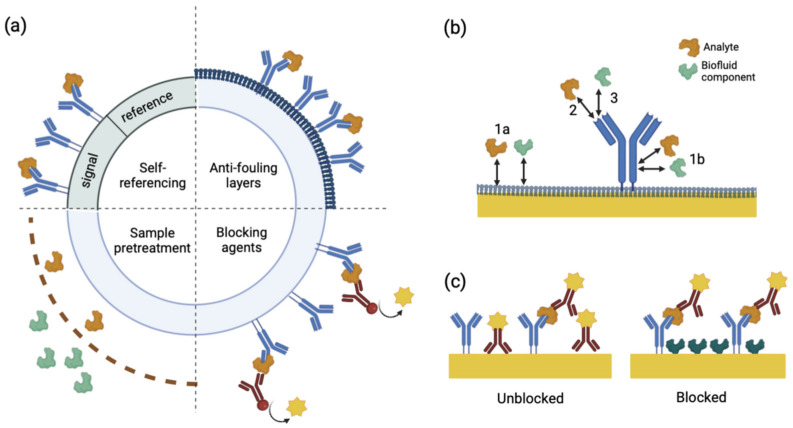
Schematic representation of nonspecific binding pathways and blocking-based prevention in biosensors. (**a**) General strategies used to minimize nonspecific binding, including self-referencing, antifouling layers, sample pre-treatment, and blocking agents. (**b**) Nonspecific binding (NSB) pathways at the sensor interface: (1a) direct adsorption onto the transducer surface, (1b) adsorption to the capture probe, and (3) interferent occupation of the recognition region, contrasted with the desired specific analyte–probe interaction (2). (**c**) Comparison of unblocked and blocked interfaces showing the reduction in nonspecific adsorption after application of blocking agents. Reproduced from Sekhon et al. [36].

The principal nonspecific binding pathways addressed by post-functionalization passivation strategies are schematically summarized in Figure 6, including direct adsorption to the transducer surface, interaction with the capture probe, and interference at the recognition site, together with the suppressive role of blocking agents.

Overall, interface passivation after receptor immobilization remains a critical second line of defence against fouling and matrix interference, complementing intrinsic antifouling materials by eliminating residual chemical and structural defects that would otherwise propagate into false-positive signals.

### 5.3. Ion-Selective Membrane Engineering

In ion-selective electrodes (ISEs), interference from competing ions is primarily caused through membrane-level selectivity engineering rather than post-measurement correction. Unlike affinity biosensors, where fouling and blocking dominate, potentiometric failure in ISEs often arises from co-extraction of interfering ions, unstable membrane phase-boundary potentials, water-layer formation, and insufficient ion discrimination [66]. The most effective prevention strategies therefore involve the rational selection of ionophores, lipophilic ionic additives, and membrane polarity, which together determine selectivity coefficients, detection limits, and long-term drift behavior.

The most classic and widely used practical example is the valinomycin-based potassium-selective membrane, where the high geometric complementarity of the ionophore cavity strongly favors K^+^ over Na^+^ and other alkali ions. However, selectivity and detection limit are further improved by introducing lipophilic ionic salts such as Potassium tetrakis(4-chlorophenyl)borate (KTpClPB) or Potassium tetrakis[3,5-bis(trifluoromethyl)phenyl]borate (KTFPB), which stabilize membrane selectivity and suppress interference-driven transmembrane ion fluxes. A foundational study by Telting-Diaz and Bakker demonstrated that tetraphenylborate-type ion exchangers directly influence lower detection limits and interference control, particularly in ultratrace analysis, by limiting undesired exchange of interfering ions at the sample side [67].

A highly practical modern example is a potassium-selective conductometric membrane sensor for sweat analysis, where a crown-ether-type potassium ionophore (K-III/BME-44) was combined with KTpClPB in a PVC membrane deposited on interdigitated gold electrodes [68]. The authors systematically showed that increasing the lipophilic salt fraction from 5 to 14 wt% reduced membrane noise, broadened the dynamic range, and improved interference suppression, although with a moderate trade-off in slope sensitivity. Most importantly, the optimized membrane matched the physiological sweat K^+^ range (1.75–16.75 mM), providing a direct real-world example of how lipophilic salts are used to manage matrix-derived ionic cross-talk in wearable ISEs. The optimized membrane composition additionally reduced membrane noise and broadened the dynamic sensing range compared with lower lipophilic salt contents. Another strong mechanistic example comes from impedance-based potassium ISEs containing valinomycin and ionic sites, where systematic variation in ionophore concentration and lipophilic borate sites was used to tune selectivity and response stability. The study showed that both the ionophore-to-site ratio and membrane thickness influence the degree of interference suppression, confirming that selectivity is not determined by the ionophore alone but by the whole membrane architecture. This concept extends beyond potassium sensing. In solid-contact coulometric ISEs for divalent cations, membranes containing calcium ionophore IV or lead ionophore IV together with KTpClPB/KTFPB were shown to improve sensitivity and reduce interference through optimized ion-exchange equilibria and stabilized charge transduction. These examples are particularly relevant for serum and environmental monitoring, where divalent ion competition is a major analytical challenge.

Recent studies have described the phenomenon of Donnan breakdown, which represents a key limiting factor for the upper detection range of ion-selective electrodes in complex real-world sample matrices [69]. They demonstrated that at high sample concentrations, the permselectivity of the ISE membrane can be overwhelmed. Importantly, no universal concentration threshold for Donnan breakdown exists, as the onset of permselectivity loss depends strongly on membrane composition, ionophore/additive ratios, membrane thickness, and the ionic composition of the sample matrix. This failure occurs when the Donnan exclusion barrier is breached, allowing interfering anions (e.g., Cl^−^ from the sample) to co-extract with cations into the organic phase, thereby disrupting the stable phase-boundary potential. Their findings emphasize that precise optimization of lipophilic salt concentration is necessary to not only achieve low detection limits but also to maintain signal stability in challenging, high-salinity biofluids such as sweat and undiluted serum.

To ensure the long-term reliability of sensors in complex environments, advanced characterization techniques are increasingly integrated into the sensor operation. Electrochemical impedance spectroscopy (EIS) has been demonstrated as a powerful diagnostic tool capable of evaluating the functionality of polymer membrane-based ion-selective electrodes (ISEs) without the need for frequent classical calibrations. By analyzing impedance characteristics, it is possible to distinguish between various types of membrane degradation, such as physical damage, biofouling, or the leaching of active components like ionophores and ionic sites. Specifically, physical damage to the membrane surface can be identified by the appearance of additional semicircles in Nyquist plots and a decrease in Warburg impedance, indicating new low-resistance pathways for ion diffusion. Furthermore, the initiation and growth of a biofilm (glycocalyx) can be monitored via EIS, as the biofilm acts as an additional diffusion layer that alters the bulk membrane resistance and interfacial charge-transfer processes [70,71]. Implementing such electronic self-diagnostics allows for the detection of critical performance thresholds, ensuring that only analytically acceptable data are reported during remote or continuous monitoring.

### 5.4. Solid-Contact Layers in ISEs

A complementary strategy to reduce interferences is the introduction of a solid-contact layer between the ion-selective membrane and the transducer in ion-selective electrodes (ISEs).

A critical long-term instability mechanism in ion-selective electrodes (ISEs) is the formation of an aqueous water layer at the interface between the ion-selective membrane and the underlying transducer surface. This film forms as a result of water uptake and transport through the membrane and acts as an uncontrolled ion reservoir, promoting slow ion exchange, potential drift, memory effects, and increased sensitivity to changes in sample composition [30,72]. The composition of the water layer may further change upon modification of the sample solution, due to the penetration of interfering ions into the layer, which contributes to additional potential instability.

The risk of water-layer formation is particularly high when hydrophilic solid-contact transducer material layers are used. To prevent this effect, effective strategies involve the introduction of hydrophobic intermediate layers, such as carbon nanomaterials or hydrophobic redox-active solid contacts. These materials reduce interfacial wetting and prevent water accumulation at the interface [30,72,73]. In practical sensor design, combining hydrophobic transducer surfaces with membrane matrices characterized by low water uptake significantly improves baseline stability and long-term potentiometric performance in complex samples such as serum, sweat, and environmental matrices.

**Figure 7:**
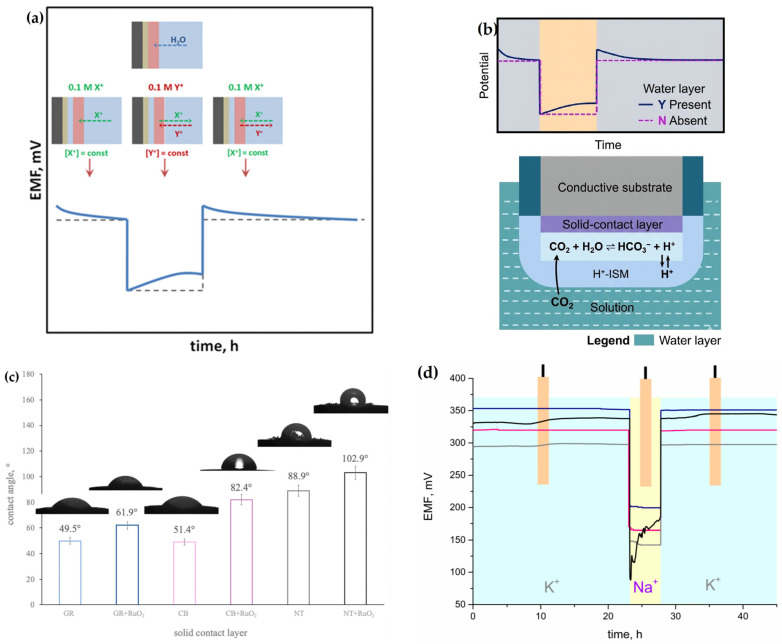
Investigation of the water layer effect and surface properties of solid-contact ion-selective electrodes. (**a**) Representative potentiometric response during a water layer test. The solid line indicates the significant potential drift characteristic of electrodes with an undesirable water layer, while the dashed line demonstrates the stability of electrodes lacking this layer. Reproduced from Wardak et al. [75]. (**b**) Schematic illustration of the interference mechanism caused by the presence of a water layer at the interface between the solid-contact layer and the ion-selective membrane (H^+^-ISM). The diagram depicts the CO_2_ transport and the resulting pH shifts that lead to potential instability. Adapted from Shao et al. [76]. (**c**) Surface wettability and experimental stability comparison for various modified glassy carbon (GC) electrodes. Surface wettability analysis of transducer materials: graphene (GR), carbon black (CB), and carbon nanotubes (NT), alongside their RuO_2_ composites. (**d**) Corresponding water layer tests for K^+^-selective electrodes: GC/CB + RuO_2_ (pink), GC/NT + RuO_2_ (grey), GC/GR + RuO_2_ (blue), and unmodified GC (black). The increased hydrophobicity of the composites (higher contact angles) directly correlates with the improved potential stability and reduced interference from Na^+^ ions. Reproduced from Lenar et al. [77].

The tendency of SC-ISEs to form a water layer is commonly evaluated using water-layer tests, typically following the procedure proposed by Fibbioli et al. [72,74]. These tests involve sequential immersion of the electrode in high-concentration solutions of the primary ion and an interfering ion (main ion → interfering ion → main ion), while monitoring potential changes, as presented in Figure 7a. Electrodes prone to water-layer formation exhibit noticeable potential drift during solution changes, caused by ion exchange within the trapped aqueous layer. In contrast, electrodes without a water layer show stable potentials after equilibration in the interfering ion solution and rapidly return to their initial potential upon re-exposure to the primary ion solution, indicating good stability and absence of interfacial water accumulation (see Figure 7b).

Lenar et al. demonstrated the beneficial effect of hydrophobic layers by employing multi-walled carbon nanotubes (MWCNTs), graphene (GR), carbon black (CB) and ruthenium dioxide–carbon nanomaterials (RuO_2_-CM) composite materials as a solid contact in ion-selective electrodes (Figure 7c). Results, obtained during the potentiometric water layer test, showed that the transition from a primary ion solution to an interfering ion solution resulted in a remarkably stable potential without the characteristic “drift” associated with water accumulation. As seen in the provided potentiometric plots, the absence of a water film at the hydrophobic carbon nanomaterial/membrane interface ensures that the potential remains constant even when the sample matrix changes significantly.

### 5.5. Regeneration, Self-Cleaning, and Reusable Sensor Interfaces

Beyond passive antifouling and post-functionalization blocking, long-term biosensing increasingly requires active regeneration of the sensing interface after target capture or fouling accumulation. Regeneration strategies aim to restore the original analytical performance by removing bound analytes, fouling layers, or exhausted receptor coatings while preserving the structural and functional integrity of the transducer [78,79,80]. This concept is particularly important in continuous monitoring, organ-on-chip systems, wearable sensors, and SPR platforms, where single-use interfaces are incompatible with prolonged operation.

**Figure 8:**
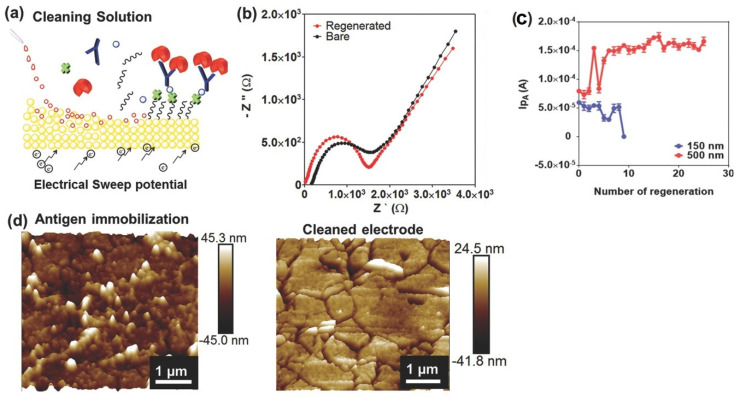
Optimization and validation of electrochemical microelectrode regeneration. (**a**) Cleaning Mechanism: Schematic representation of the regeneration process using a cleaning solution combined with an electrical sweep potential to remove immobilized biolayers. (**b**) Electrochemical Verification: Nyquist plots comparing a bare electrode (black) and a regenerated electrode (red). The overlapping semi-circles demonstrate the successful restoration of the charge-transfer resistance (R_ct_) after the cleaning cycle. (**c**) Reusability and Durability: Evaluation of the regeneration efficiency over multiple cycles. The peak current (IpA) stability indicates that thicker gold layers (500 nm, red) sustain significantly more regeneration cycles compared to thinner layers (150 nm, blue), defining the functional lifespan of the reusable sensor interface. (**d**) Surface Morphology: Atomic Force Microscopy (AFM) images illustrating the microelectrode surface (**right**) following antigen immobilization and (**left**) after the regeneration protocol, showing the removal of biological material and restoration of surface topography. Reproduced from Shin et al. [81].

An example of electrochemical re-functionalization after use was demonstrated by label-free regenerative electrochemical microfluidic biosensors for continual monitoring of cell secretomes, targeting creatine kinase-MB (CK-MB) in organ-on-chip culture media (Figure 8) [81]. The system integrated a microfluidic EIS biosensor with automated two-step regeneration using cyclic voltammetric cleaning in H_2_SO_4_ followed by K_3_Fe(CN)_6_ treatment, enabling removal of the complete SAM–bioreceptor architecture from gold microelectrodes. The cleaned interface was then re-functionalized in-line, allowing repeated biomarker measurements over multiple cycles.

In optical biosensors, chemical regeneration of affinity interfaces remains one of the most established approaches. In SPR systems, regeneration is typically achieved using low-pH glycine buffers, high-salt eluents, surfactants, or chaotropic solutions, which dissociate antigen–antibody or aptamer–target complexes without stripping the immobilized ligand [78]. Recent clinical SPR reviews highlight regeneration as a critical requirement for translating SPR into routine diagnostics, especially for repeated biomarker measurements in serum and plasma [78]. In practice, this is particularly important for microfluidic SPR chips, where regeneration efficiency directly determines throughput, cost per assay, and baseline reproducibility. A highly modern direction involves regeneration through receptor-level design, particularly in aptamer and nucleic acid biosensors. Jia et al. summarized multiple examples where chemical, thermal, or pH-induced conformational resetting of aptamers restores target-free receptor states without replacing the sensing surface [79]. These strategies are especially attractive because regeneration occurs at the level of the recognition event itself, rather than by rebuilding the entire sensor architecture. Such approaches are promising for cytokine, nucleic acid, and metabolite biosensors in sweat, saliva, and interstitial fluid.

Another important strategy is electric-field-driven or electrochemical self-cleaning, particularly for MIP and electroactive biosensors. In these systems, the application of potential pulses, cyclic voltammetry, or controlled over-oxidation can desorb electroactive foulants, release captured analytes, or re-open imprinted cavities [80,81]. Representative examples include wearable laser-engraved graphene MIP sensors for amino acids and cortisol, where electrical stimulation enabled repeated regeneration cycles without external washing steps. This approach is especially promising for wearable devices because it supports in situ cleaning during continuous biofluid exposure.

From a broader design perspective, regeneration strategies can be grouped into three major categories:

- surface stripping and re-functionalization (electrochemical microchips);
- target dissociation with preserved receptors (SPR, aptamers);
- stimuli-responsive self-cleaning interfaces (electric, pH, thermal, light).

Each strategy offers a different balance between regeneration efficiency, transducer preservation, and cycle lifetime.

To address the challenges of signal degradation in wearable sweat diagnostics, Hou et al. [82] recently developed a hierarchical sensing platform that integrates passive antifouling with active self-cleaning capabilities for the continuous monitoring of uric acid (UA). The architecture consists of a dual-layer interface: a top TiO_2_/PVDF nanofiltration membrane and a bottom rGO/polypeptide hydrogel sensing layer. The TiO_2_/PVDF membrane serves as a physical barrier that selectively excludes large-scale contaminants such as shed skin keratinocytes while maintaining a superhydrophilic surface to repel sebaceous oils. To address smaller molecular interferents, the underlying zwitterionic hydrogel creates a dense hydration shell that prevents the non-specific adsorption of proteins and bacteria. This in situ regeneration strategy allows the sensor to recover its initial sensitivity and surface cleanliness without requiring manual intervention, providing a robust model for the development of long-term, reusable wearable interfaces in complex biological fluids.

Despite their significant promise for long-term biosensing, stimuli-responsive and actively regenerated interfaces also raise important biocompatibility considerations for future in vivo and implantable applications. Electrically stimulated self-cleaning systems, conductive polymers, and responsive hydrogel architectures may potentially induce local inflammatory responses, cytotoxicity, or tissue irritation depending on their composition, degradation products, and operational conditions. In addition, repeated electrochemical stimulation or oxidative cleaning procedures may affect long-term tissue compatibility and interfacial stability in subdermal environments. Consequently, future development of self-cleaning biosensors will require careful optimization of material biocompatibility, degradation behavior, and stimulation parameters before clinical implantation can be safely achieved [83].

### 5.6. Sample Pre-Treatment and Microfluidics

A highly effective way to mitigate fouling and matrix interference is to reduce the complexity of the sample before it contacts the sensing interface. Unlike surface-level antifouling approaches, microfluidic pre-treatment acts upstream by removing cells, large proteins, lipids, vesicles, and viscosity-related interferents that would otherwise destabilize the transducer response. This strategy is particularly important for whole blood, plasma-rich protein assays, and continuous sensing workflows, where direct exposure of the sensing interface to untreated samples often leads to rapid blocking, hemolysis artifacts, and poor reproducibility [84,85,86,87].

**Figure 9:**
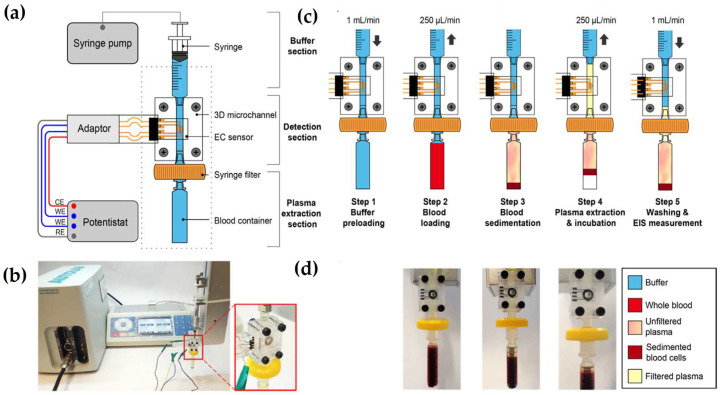
Integrated microfluidic platform for automated blood pre-treatment and in situ biosensing. (**a**,**b**) Schematic layout and photographic representation of the microfluidic device, highlighting the embedded electrochemical (EC) sensor and the fluidic network connecting the sample inlet and buffer reservoirs. (**c**) Operational Workflow: The five-step on-chip protocol involving (1) buffer preloading, (2) whole blood injection, (3) gravitational sedimentation for blood cell separation, (4) plasma extraction and target analyte incubation, and (5) surface washing followed by electrochemical impedance spectroscopy (EIS) analysis. (**d**) Visualization of On-Chip Processing: Time-sequenced images demonstrating the successful transition from whole blood loading to passive sedimentation and the final extraction of clear plasma into the sensing chamber. Reproduced from Lai et al. [84].

A notable example is the platform developed by Lai et al. [84], which utilizes a sedimentation-based microfluidic design to extract plasma from whole blood without the need for external centrifugation. As illustrated in Figure 9, the device guides the sample through a sequential protocol, including buffer preloading, gravitational sedimentation of blood cells, and subsequent plasma incubation, before performing in situ electrochemical impedance spectroscopy (EIS). This integration not only reduces the total analysis time but also minimizes the matrix interference of cellular components, demonstrating the potential for ‘sample-to-answer’ systems in point-of-care diagnostics.

A second highly relevant strategy is paper-based microfluidic blood plasma separation (µPAD) integrated with downstream biomarker analysis. Burgos-Flórez et al. developed a microfluidic paper-based blood plasma separation device for S100B detection, a clinically relevant traumatic brain injury biomarker, using whole blood as the starting matrix [85]. The device generated approximately 50 µL of plasma from 300 µL of blood within 3.5 min, allowing downstream immunodetection while minimizing cellular interference and hemolysis-derived artifacts. This work is particularly valuable because it shows that low-cost passive filtration can function as a true antifouling pre-treatment step in POC diagnostics.

More advanced fluidic control strategies use sample-to-answer microchips with integrated plasma separation and dilution modules. In a strong Lab on a Chip example, Kikkeri et al. integrated a commercial Vivid membrane for blood-to-plasma separation at the front-end of an electrochemical biosensor system, using IL-6 as a model biomarker [87]. Importantly, the authors did not stop at cell removal; they also optimized dilution ratios to minimize plasma matrix effects, directly addressing viscosity, protein concentration, and nonspecific adsorption. This makes the study an excellent example of how filtration and controlled dilution jointly mitigate both fouling and bulk-matrix distortions.

### 5.7. Data Correction and Drift Compensation

Despite significant advances in antifouling materials, interface engineering, and microfluidic pre-treatment, complete elimination of fouling and matrix interference remains unrealistic in complex biological environments. As a result, increasing attention has been directed toward algorithmic and data-driven strategies that compensate for signal distortion at the level of data processing rather than interface design [88,89]. These approaches are particularly valuable in continuous monitoring systems, wearable devices, and multiplexed biosensors, where baseline drift, noise, and matrix variability cannot be fully controlled physically.

One of the most widely implemented approaches is baseline drift correction using signal processing and calibration models. For example, in continuous electrochemical sensing platforms, adaptive baseline tracking and filtering algorithms have been used to compensate for slow signal drift caused by protein adsorption, electrode aging, and electrolyte fluctuations [88]. Such methods are especially important in wearable sensors operating in sweat or interstitial fluid, where hydration changes and ionic strength variations continuously perturb the signal.

Machine learning (ML) approaches further extend this concept by enabling pattern recognition in complex, multidimensional sensor outputs. In a notable example, Zhang et al. developed a machine-learning-assisted electrochemical sensing system capable of distinguishing target signals from interfering species in complex biofluids, improving analytical accuracy without modifying the sensing interface itself [90]. Similar approaches using neural networks and regression models have been applied to multi-analyte sensor arrays to separate overlapping signals from species such as ascorbic acid, uric acid, and glucose.

In optical biosensing, particularly SPR, reference channel subtraction and real-time signal normalization algorithms are routinely used to compensate for bulk refractive index fluctuations and nonspecific adsorption [89]. Advanced implementations combine dual-channel referencing, temperature compensation, and flow-rate normalization, allowing reliable biomarker detection even under dynamically changing sample conditions. These strategies are essential because optical systems are inherently sensitive to bulk matrix variations that cannot be fully eliminated by surface chemistry alone.

A more advanced concept is the use of digital twins for biosensing systems. A digital twin is a real-time computational model that runs in parallel with the physical sensor. It simulates mass transport, interfacial reactions, and environmental effects such as pH and temperature. By continuously comparing simulated and experimental signals, digital twins can distinguish true analyte changes from drift caused by fouling or sensor aging, enabling real-time correction and predictive calibration [91].

To facilitate practical translation of the discussed prevention concepts into sensor design workflows, the major antifouling, blocking, regeneration, pre-treatment, and algorithmic compensation strategies are comparatively summarized in Table 3.

## 6. Future Perspectives

Despite significant advances in antifouling strategies, interface engineering, and data-driven correction methods, matrix effects and fouling-related signal instability remain one of the principal barriers to the long-term reliability of sensors and biosensors in real biological environments. Future developments are therefore expected to move beyond static antifouling coatings toward dynamic, adaptive, and self-regulating sensing interfaces capable of responding to complex and evolving biological matrices [21,41].

A major emerging direction is the development of self-cleaning and self-regenerating interfaces, which aim to actively remove or reorganize adsorbed foulants without external intervention. These systems include stimuli-responsive polymers, electrochemically switchable surfaces, and photoactive coatings capable of triggering localized desorption of proteins and biomolecular aggregates. Such approaches are particularly promising for continuous and wearable biosensors, where long-term exposure to sweat, interstitial fluid, or serum-like environments leads to progressive fouling and baseline drift [41].

Closely related are smart regenerable sensing surfaces, which combine antifouling functionality with reversible binding architectures. These systems are designed to maintain analytical performance over multiple measurement cycles through controlled disruption of nonspecific adsorption layers while preserving the integrity of biorecognition elements. In optical and electrochemical platforms, regeneration is increasingly being integrated at the material design level rather than treated as an external post-processing step.

Another rapidly growing research direction involves anti-biofilm and anti-fouling molecular design, inspired by natural non-fouling surfaces and biological defense mechanisms. Zwitterionic chemistries, highly hydrated polymer networks, peptide-mimetic coatings, and biomolecular crowding-resistant architectures are being explored to suppress both initial protein adsorption and subsequent biofilm formation. These strategies are particularly relevant for implanted and epidermal biosensors, where long-term exposure to biological fluids promotes progressive microbial and protein accumulation.

In parallel, data-driven approaches are expected to play an increasingly central role in mitigating matrix-induced signal distortion. Machine learning models for drift prediction, adaptive baseline correction, and real-time artifact classification are emerging as powerful tools for compensating for unavoidable interfacial instability. These approaches enable sensors to operate under non-ideal conditions by distinguishing true analyte signals from fouling- or matrix-induced variability [21].

A particularly promising concept is the development of digital twins for sensor systems, in which a computational replica of the sensor interface continuously simulates expected performance under varying biological and environmental conditions. Such models can incorporate fouling kinetics, mass transport limitations, and electrochemical or optical response dynamics to predict sensor aging and guide real-time calibration strategies. This paradigm may enable predictive maintenance of biosensing devices in clinical and wearable applications.

Finally, future biomarker sensing platforms are likely to rely on hybrid multimodal confirmation systems, integrating electrochemical, optical, mechanical, or thermal transduction within a single analytical platform. By cross-validating signals from independent sensing modalities, these systems can significantly reduce false-positive and false-negative rates arising from matrix interference. Such multimodal architectures are particularly attractive for point-of-care diagnostics, where robustness and reliability are more critical than single-mode sensitivity.

## 7. Conclusions

The performance of sensors and biosensors in biological samples is fundamentally limited by the combined effects of fouling, surface blocking, and matrix interference. These processes are strongly interconnected and collectively lead to signal drift, reduced sensitivity, and poor analytical reliability, particularly in complex media such as serum or whole blood.

This review emphasizes that sensor failure originates at the interface, where nonspecific adsorption, electroactive species, and physicochemical fluctuations alter both recognition and signal transduction. Because these effects occur simultaneously, effective prevention cannot rely on a single strategy. Instead, robust sensing requires an integrated approach combining antifouling materials, surface passivation, transducer-specific optimization, and sample pre-treatment.

In addition, data-driven methods such as drift correction and machine learning are becoming increasingly important for compensating residual matrix effects. Future progress will depend on the development of adaptive and self-regenerating interfaces, as well as the integration of microfluidics and intelligent data processing.

Overall, improving sensor reliability in real biological environments requires a coordinated strategy that links surface chemistry, device engineering, and computational analysis.

## Figures and Tables

**Table 1:** Comparison of fouling-related mechanisms in biosensors and chemical sensors. Overview of biofouling, surface blocking, and matrix interference effects across biosensors and chemical sensors.

Mechanism	Biosensors	Chemical Sensors
Biofouling	passivates receptor layer	blocks surface
Surface blocking	masks binding sites	reduces active area
Matrix interference	affects binding affinity	affects signal physics

**Table 2:** Transducer-specific failure mechanisms in biological matrices.

Sensor Type	Main Bio-Interferents	Failure Mechanism	Key Analytical Problems
Potentiometric (ISE)	Proteins (adsorption), lipid fragments, fluctuating electrolyte ions (K, Na, Cl).	Perturbation of phase-boundary equilibrium; formation of water layers at solid contacts.	Baseline drift, loss of Nernstian slope, and sensitivity to junction potentials.
Voltammetric/Amperometric	Uric acid, ascorbic acid, and protein-based “fouling” layers.	Electrode surface passivation; competitive oxidation/reduction in small molecules.	Reduced electron-transfer efficiency, suppressed current peaks, and false-positive faradaic signals.
Impedimetric/Capacitive (EIS)	Serum albumin, immunoglobulins, and extracellular vesicles.	Nonspecific increase in charge-transfer resistance or change in dielectric permittivity.	False-positive binding signals; similar impedance changes from fouling
Optical/Plasmonic (SPR/LSPR)	High-abundance proteins, lipoproteins, and hemolysis products (hemoglobin).	Nonspecific mass loading and bulk refractive index (RI) fluctuations.	Resonance angle/wavelength shifts, baseline elevation, and incomplete surface regeneration.
Field-Effect Transistor (BioFET)	High ionic strength fluids (serum/sweat), mobile ions, and charged protein layers.	Electrostatic charge screening (Debye effect) and threshold voltage drift.	Severe signal loss (shielding) and ionic instability at the semiconductor gate.

**Table 3:** Practical prevention guidelines summarizing Section 5: from antifouling interface design to computational drift correction.

Prevention Strategy	Main Mechanism	Best Suited Sensor Types	Ref.
Hydration-based antifouling (hydrogels, PEG, zwitterions)	Formation of hydrated barrier that suppresses nonspecific adsorption while allowing analyte diffusion	Electrochemical, wearable, BioFET	[43,44,45,46,47,48]
Conductive hydrogels	Combined antifouling, charge transport, and signal amplification in hydrated 3D matrix	Electrochemical, wearable	[48]
Polymer brushes (PEG, OEGMA, carboxybetaine)	Dense surface-grafted chains create steric and hydration repulsion against proteins and bacteria	SPR, electrochemical, BioFET	[52,53]
Self-assembled monolayers (SAMs)	Ordered molecular packing reduces surface accessibility and nonspecific adsorption	Electrochemical, SPR	[49]
Electrosynthesized permselective polymer coatings	Electrostatic and size-exclusion suppression of electroactive interferents	Amperometric biosensors	[54,55,56]
ISE antifouling coatings (hydrophilic/low-adhesion layers)	Reduction in membrane-tip fouling and diffusion barriers to stabilize potentiometric response	Ion-selective electrodes (ISEs), wearable sensors	[59]
Post-functionalization blocking (ethanolamine, MCH, BSA)	Chemical deactivation of reactive sites, defect filling, and suppression of nonspecific binding	Electrochemical, SPR, immunosensors, aptasensors	[60,61,62,63,64]
Membrane selectivity engineering (ISEs)	Ionophore–ion specificity and lipophilic salt control of ion exchange and interference	Ion-selective electrodes	[67,68]
Donnan equilibrium control	Prevention of co-extraction of interfering ions at high concentration	Ion-selective electrodes	[69]
Solid-contact engineering (hydrophobic layers)	Elimination of water-layer formation and interfacial ion reservoir	Solid-contact ISEs	[30,72,73,74,75,76,77]
Regeneration/self-cleaning	Removal and rebuilding of sensing interface after fouling or use	Electrochemical, SPR, wearable, microfluidics	[78,79,80,81,82]
Electrochemical self-cleaning	Chemical, electrochemical, or stimuli-driven removal of fouling and bound targets; surface reuse	Wearable electrochemical sensors	[80]
Microfluidic sample pre-treatment	Removal of cells, proteins, and interferents before sensing	Lab-on-chip, electrochemical	[84]
Paper-based plasma separation (µPAD)	Passive filtration enabling low-cost antifouling pre-treatment	Point-of-care, paper-based sensors	[85]
Filtration + dilution microfluidics	Combined removal of interferents and matrix normalization	Lab-on-chip, electrochemical	[87]
Baseline drift correction (algorithms)	Signal processing to compensate time-dependent drift	Wearable, continuous monitoring	[88]
Machine learning-assisted sensing	Pattern recognition to separate signal from interference	Electrochemical sensors	[90]
Digital twins	Real-time modeling to distinguish drift vs. analyte signal	Advanced biosensing systems	[91]

## Data Availability

No new data were created or analyzed in this study. Data sharing is not applicable to this article.

## Abbreviations

The following abbreviations are used in this manuscript:

AI	Artificial Intelligence
BioFET	Field-Effect Transistor Biosensor
BSA	Bovine Serum Albumin
CB	Carbon Black
EDC	1-ethyl-3-(3-dimethylaminopropyl)carbodiimide
EIS	Electrochemical Impedance Spectroscopy
GC	Glassy Carbon
GR	Graphene
HDL	High-Density Lipoprotein
ISE	Ion-Selective Electrode
LDL	Low-Density Lipoprotein
LSPR	Localized Surface Plasmon Resonance
MCH	6-mercapto-1-hexanol
NHS	N-hydroxysuccinimide
NT	Carbon Nanotubes
OEGMA	Oligo(ethylene glycol) methacrylate
PEG	Poly(ethylene glycol)
POC	Point-of-Care
RI	Refractive Index
SAM	Self-Assembled Monolayer
SPR	Surface Plasmon Resonance
